# A pilot randomised controlled trial of the Spinal Cord Injury and You (SCI&U) online peer health coaching self-management program

**DOI:** 10.1186/s40814-026-01769-y

**Published:** 2026-01-28

**Authors:** Susan B. Jaglal, Sonya J. Allin, B. Catharine Craven, Sara J. T. Guilcher, A. Gary Linassi, Christopher B. McBride, Rahim Moineddin, W. Ben Mortenson, Sarah Munce, Nancy M. Salbach, John D. Shepherd, Shane N. Sweet, Teri Thorson, Jennifer R. Tomasone

**Affiliations:** 1 Department of Physical Therapy, University of Toronto, Toronto, ON Canada; 2 Rehabilitation Sciences Institute, University of Toronto, Toronto, ON Canada; 3 Institute of Health Policy, Management and Evaluation, University of Toronto, Toronto, ON Canada; 4 KITE Research Institute, University Health Network, Toronto, ON Canada; 5 Department of Electrical Engineering and Computer Science, York University, Toronto, ON Canada; 6 Division of Physical Medicine and Rehabilitation, University of Toronto, Toronto, ON Canada; 7 Leslie Dan Faculty of Pharmacy, University of Toronto, Toronto, ON Canada; 8 Department of Physical Medicine and Rehabilitation, University of Saskatchewan, Saskatoon, SK Canada; 9 Spinal Cord Injury BC, Vancouver, BC Canada; 10 Department of Family & Community Medicine, University of Toronto, Toronto, ON Canada; 11 Dalla Lana School of Public Health, University of Toronto, Toronto, ON Canada; 12 Department of Occupational Science and Occupational Therapy, Faculty of Medicine, University of British Columbia, Vancouver, BC Canada; 13 International Collaboration on Repair Discoveries, Vancouver General Hospital, Vancouver, BC Canada; 14 Bloorview Research Institute, Holland Bloorview Kids Rehabilitation Hospital, Toronto, ON Canada; 15 Department of Kinesiology and Physical Education, McGill University, Montreal, QC Canada; 16 Centre for Interdisciplinary Research in Rehabilitation of Greater Montreal, Montreal, QC Canada; 17 School of Kinesiology and Health Studies, Queen’s University, Kingston, ON Canada

**Keywords:** Self-management, Spinal cord injuries, Peer group, Pilot study, Internet-based intervention

## Abstract

**Background:**

The Spinal Cord Injury and You (SCI&U) intervention aims to improve self-management skills for persons living with SCI using a web-based, peer health-coaching model. This study assessed feasibility of a future definitive trial of SCI&U, specifically feasibility of recruitment and retention, program usability and quality, effect size estimates for self-management outcomes and rehospitalisation rates (i.e. health-related quality of life).

**Methods:**

A two-group, randomised, controlled, pilot trial with prospective recruitment, concealed group allocation, blinded outcome evaluation and waitlist control was conducted. We aimed to recruit 60 adult participants living in the community at least 6 months post-injury who could speak and read English and had a family physician. The intervention included up to 14 1-h online client-coach videoconferencing sessions, goal setting, action planning and a sortable resource library. Data were collected at baseline, 2, 6 and 12 months post-randomisation. SCI&U was offered to waitlist participants at 12 months.

**Results:**

Trial methodology and procedures were feasible. Recruitment and retention targets were achieved. Individuals were randomised to intervention (*n* = 31) and waitlist control (*n* = 34). Mean time since SCI was 25.6 years (intervention) and 20.2 years (control). Timeline for completion of online sessions was extended from 2 months to 6 months. Outcome data were gathered for 86% (6 months) and 89% (12 months) of participants. Program usability and quality were highly rated on the Mobile App Rating Scale. Difference in Skill and Technique Acquisition subscale between intervention and control was 0.56 (95%CI -0.41, 1.52) at 6 months and 0.72 (95%CI -0.28, 1.72) at 12 months. Other Health Education Impact Questionnaire subscales had large effect sizes: self-monitoring and insight 1.51 (95% CI 0.39, 2.69); emotional distress -1.40 (95%CI -3.04, 0.23). In 12 months post-recruitment, 5 intervention and 4 control participants spent median 11 (95% CI 3-19) and 24 (95% CI 5-95) nights in hospital, respectively.

**Conclusions:**

Trial methodology and procedures were feasible. The SCI&U intervention was acceptable to participants and positively impacted an individual’s ability to self-manage. A definitive trial is warranted to build on these findings, particularly in those with recently acquired SCI. Future recruitment efforts will focus on inpatient rehabilitation hospitals to recruit individuals < 5 years post-injury.

**Trial registration:**

ClinicalTrials.gov, NCT04474171, retrospectively registered 07/13/2020; https://clinicaltrials.gov/study/NCT04474171#study-record-dates.

## Key messages regarding feasibility

- Feasibility of participant recruitment, participant retention with long-term follow-up, data collection and implementation of the SCI&U online peer health coaching program were assessed, as well as their effects on program usability and quality.
- Overall, the trial methodology and procedures were feasible, and the intervention was acceptable to participants. Recruitment and retention targets for the program were achieved, even during the COVID-19 pandemic. However, it was difficult to recruit individuals with recent SCI, such as within five years of injury. Also, there were too many outcome measures to complete.
- Changes to improve delivery of the intervention and trial methodology in the definitive trial include: focus recruitment efforts on inpatient rehabilitation hospitals to recruit individuals who are newly injured; limit outcome measures to only those that showed potential for change; and extend the timeline for completion of online sessions from 2 to 6 months.

## Background

Living with the consequences of spinal cord injury (SCI) is a life-long process [1], beyond the initial trauma and adjustment to impairments to body systems and functions (e.g. sensory, bowel and bladder) and activity limitations (e.g. mobility, self-care and interpersonal relationships). SCI results in a variety of acute motor, sensory and autonomic impairments typically requiring tertiary care and rehabilitation to optimize patient outcomes. After discharge from inpatient rehabilitation, individuals with SCI continue to be predisposed to multiple impairments and have an increased propensity for secondary health complications [2–4]. In the first year post-injury, over 50% of people discharged with an SCI may require rehospitalisation due to a secondary complication, such as a urinary tract infection, pressure ulcer or pneumonia [5, 6]. One-year rehospitalisation rates in Canada have remained high, at over 27%, for more than 10 years [7]. At 10 years post-injury, patients with SCI report a mean of 7 secondary health conditions [4], and health-related quality of life and health utility scores are well below other vulnerable populations [8]. Even 20 years post-injury, rehospitalisation rates remain over 30% due to new functional declines and complications from aging, such as cardiovascular disease, diabetes, bone mineral density loss, fatigue and respiratory complications or infections [4–6].

Length of stay in inpatient rehabilitation has decreased dramatically [9, 10]. The limited time for provision of health information and skill acquisition in the inpatient rehabilitation setting due to the shorter length of stay means individuals with SCI are entering the community with fewer self-management skills to prevent secondary complications [11, 12]. Families and others comprising informal support networks also have less time to adjust [13]. Individuals with SCI report that their primary care providers are not well-equipped to support their specialized needs [1, 14, 15], leading to higher rates of secondary complications, emergency department visits and rehospitalisation [7, 16, 17]. Health care systems, both locally and globally, often lack inclusivity, particularly for people with a wide range of disabilities [18].The result is a growing demand to provide appropriate health information, skills and support for persons with SCI who are living in the community to better manage their health conditions across the lifespan.

Self-management programs are designed to increase the knowledge and skills required by an individual living with a chronic condition to manage symptoms, treatment, physical and psychosocial consequences and reduce the risk of secondary complications [19]. Programs such as Stanford’s Chronic Disease Self-Management Program (CDSMP) [20] or the UK’s Expert Patient Program [21], comprised of peer-led health coaching and patient education [22], are associated with improved self-efficacy, health behaviours and psychological health status [20–22], lower hospitalisation rates [20] and reduced health care expenditures [23]. Despite these positive results, a qualitative study on the experiences of CDSMP participants with neurological conditions found participants with SCI reported the least program satisfaction and thus recommended a SCI-specific program [24]. These findings were supported in our previous research wherein individuals with SCI and other knowledge users (family members/caregivers, health care professionals, consumer organizations and policy makers) emphasized the need for an SCI-specific online program led by peers [25–29].

Only three methodologically rigorous studies of two peer-led, self-management programs for SCI have been published. A randomised controlled trial (RCT) [30] and an interrupted time series analysis [31] evaluated one program developed in Atlanta, Georgia, and another RCT evaluated a program developed in Boston, Massachusetts [32]. Both programs demonstrated the value of peer-delivered interventions in improving self-management in persons with SCI, but neither of these interventions was delivered virtually; one was in person in a rehabilitation hospital [30, 31] and the other was telephone-based [32].

This gap led to the development of our online, peer-led, self-management program for SCI (SCI&U), which was identified as the preferred format in our earlier work. The Spinal Cord Injury and You (SCI&U) platform contains an integrated set of tools to support secure one-on-one online health coaching and tools to promote discussion of and access to health education resources [33, 34]. During sessions, peer health coaches help participants frame self-management goals, create action plans, and solve problems related to their health. Peer coaches are trained to provide self-management education and support to others with SCI, which is an expanded application of SCI peer mentoring [35]. The main goal of the program is to improve self-management skills among persons living with SCI.

Web-based programming is preferred over in-person programming by individuals with SCI [24, 25]. The online format simulates face-to-face interaction and limits the need for in-person visits among a population with mobility challenges [33, 34]. Internet-based programming for a nursing intervention following inpatient rehabilitation of persons with SCI was associated with fewer post-discharge hospital visits compared to phone-based interventions in a randomised trial [36].

This study reports a randomised, controlled, pilot trial of the SCI&U intervention. The primary objective was to evaluate feasibility of the program, specifically: participant recruitment; participant retention with long-term follow-up; data collection; program implementation; and participants’ assessment of usability and quality of the program. The SCI&U intervention was evaluated for: (a) primary outcomes of self-management skills and total days rehospitalised; and (b) secondary outcomes of secondary health conditions, self-efficacy, health-related quality-of-life (HRQOL) and social/role activities limitations. Effect sizes for short-term (baseline to 2 months) and sustained (6 and 12 months) impacts of the program on these outcomes were estimated to evaluate the potential benefit of the program.

## Methods

### Trial design

A two-group, randomised, controlled, pilot trial with prospective recruitment, concealed group allocation, blinded outcome evaluation and waitlist control was conducted in Canada from January 2018 to March 2022. Research Ethics Board approval was obtained at the main coordinating site University of Toronto (Protocol Number 34808) and also at the University of Saskatchewan (Protocol Number 1228). All recruited individuals formally consented to participate in this study orally with a member of the research staff, who signed and dated a paper copy retained at the research office. For consenting individuals, outcome data were gathered from questionnaires administered at baseline, and 2, 6 and 12 months after baseline. The trial was registered retrospectively on ClinicalTrials.gov (NCT04474171; 07/13/2020; https://clinicaltrials.gov/study/NCT04474171#study-record-dates). The CONSORT (Consolidated Standards of Reporting Trials) 2010 statement: extension to randomised pilot and feasibility trials [37] and the TIDieR checklist [38] were followed.

### Eligibility and recruitment of participants

We targeted individuals 18 years and older living in the community (i.e. not institutionalized in a long-term care facility or nursing home) who were at least 6 months post-injury, were able to speak and read English and had a primary care physician. Potential participants self-assessed their ability to speak and read English. During the screening interview the research assistant provided details about the intervention and data collection measures and noted all materials and the coaching sessions would be in English. Individuals who were currently participating in another formal self-management program or had a self-report of physician-diagnosed concurrent traumatic brain injury were excluded.

Recruitment for participants was nationwide with a focus on British Columbia (BC) and Ontario, where the research team had relationships with community-based SCI peer organizations, peer health coaches and research staff. Various methods were used for recruitment, including outreach by the SCI BC Peer Recruitment Coordinator, and advertisements by SCI consumer organizations on their websites, in newsletters, magazines, Facebook groups and via webinars to their members. Recruitment information was also placed on SCI&U social media accounts. Study co-investigators who worked at rehabilitation hospitals also informed clinicians about the study.

Individuals interested in the study were asked to email the coordinating centre. A research assistant contacted the potential participant, screened them for eligibility and obtained their consent to be randomised to the intervention or waitlist control group. Participants received $300 CAD if they completed all study procedures.

### Intervention

The development, usability and pilot testing of SCI&U is published elsewhere [33, 34]. We followed the mHealth framework [39] and a participatory design approach [40], consistent with the Integrated Knowledge Translation Guiding Principles for Conducting SCI research in partnership [41]. The SCI&U digital platform prototype has a resource library, tools to support one-on-one health coaching with profiles of coaches (to facilitate matching of coaches with clients) and a structured interface for health coaching. Its major features include:

1. The ability to create and schedule secure ‘themed’ videoconferencing sessions between coaches and clients. Themes (such as exercise and nutrition) dictate the scripts used to guide each session, as well as session-specific resources and self-care tips.
2. Goal setting and action planning forms. These record the goals and plans of clients as they are articulated during sessions.
3. A sortable resource library, containing themed educational material and resources with links to external websites and videos.
4. The ability for coaches and administrators to create and send customized reminders and emails to clients (e.g. session summaries).

Support materials were housed in a web-based, curated, information resource platform accessible to participants and coaches. Confidential one-on-one interactions between participants and peer health coaches took place on a specially designed secure platform [34].

### Implementation

Following randomisation to SCI&U, participants were scheduled for a registration and orientation session to become better acquainted with the platform’s features and to troubleshoot any accessibility issues; participants were offered a Chromebook or tablet on loan if needed. Participants were then partnered with their health coach, who was over age 18 and had lived in the community with SCI for more than 5 years, the amount of time perceived as needed to adapt to the initial injury [42, 43].

Coaches had three principal roles: role model, supporter and advisor [35]. Peer health coaches were recruited through a nationwide campaign involving community partners. A recruitment committee reviewed candidate applications for evidence of communication skills; passion about healthy living and helping others; understanding of SCI and self-management; competence with technology; and responsibility and resourcefulness. Candidates who were selected for an online interview with a panel of three team members were scored on their answers to open-ended questions and their perceived communication and interpersonal skills. Candidates with the highest scores were offered positions as peer health coaches. To ensure consistency and quality of the intervention, all coaches were trained in Motivational Interviewing [44] and certified in Brief Action Planning (BAP) [45] by the Centre for Collaboration, Motivation and Innovation. Further in-house training covered the theoretical background; coaching and communication tools; the coaching workflow; and the custom IT platform. Peer health coaches were compensated for their preparation time, time to deliver the intervention and for meetings with the coach coordinator and coach training.

In the first videoconference session, which took place within 1 month following consent, participants identified priority issues related to their health. In subsequent sessions, they worked through goal setting, conducted problem solving activities and created action plans for behaviour change that were securely stored by the interface and available to users to reflect upon and revise. Each online session conformed to a script; scripts were accessible to coaches via the online platform and included health management information drawn from guidelines and standardized protocols for BAP. Themes for sessions related to common health management concerns among the SCI population as indicated by a prior survey of the Canadian SCI community [25, 26] (Table 1). Coaches could take notes about clients during sessions, recommend relevant online resources and arrange follow-up care plans (e.g. send text message reminders of client goals or plans periodically).

**Table 1:** List of self-management health coaching topics

Aging Autonomic Dysreflexia Bladder Management Bone Health Bowel Management Building Your Healthcare Team Cannabis Communication with Health Care Professionals COVID-19: What is it? COVID-19: Stress and Resilience COVID-19: Respiratory Care	Diet and Nutrition Evaluating Health Information Fatigue Goal Setting Incomplete SCI Leisure and Recreation Mobility Devices Pain Management Parenting and Fertility Parenting Older Children	Physical Activity Problem Solving Relationships Skin Management Self-Advocacy Sexuality Shoulder Health Spasticity Stress, Anxiety, and Depression Women's Health

During the program, clients and coaches could engage in up to 14 online sessions, similar to two effective, telephone-based, health behaviour change interventions in SCI: Get in Motion (GIM) [46]; and My Care My Call (MCMC) [32]. We planned to implement the online sessions over 6 months with a tapered schedule (i.e. 8 weekly, 4 biweekly and 2 monthly sessions) to gradually transition clients from dependency on the coach to independent self-regulation, comparable to GIM, in which clients reported significant increases in time spent in physical activity between baseline and 2 months that was maintained at 6 months [46].

### Control

The control group continued with their usual health care, including outpatient visits to primary care physicians and rehabilitation specialists, and home care services [12]. They were offered the SCI&U program at the end of the 12-month assessment period (wait-list control).

### Outcomes

#### Trial feasibility

Feasibility of recruitment was assessed by the number and proportion of consenting individuals per month in an 8-month recruitment period. Feasibility of data collection was evaluated as percentage of participants with complete data on each measure at each evaluation time point, with targets of >90% for baseline and 2-month and >80% for 6-month and 12-month evaluations [34]. In RCTs of peer-led interventions for SCI [30, 32], the loss to follow-up at 6 months was 13% [30] and 10% [32]. Adherence was calculated as percentage of coaching sessions that participants attended. Participants completing eight or more sessions were considered adherent based on GIM study findings to increase physical activity in those with SCI [46] and the MCMC study to improve self-management to prevent secondary conditions [32]. Findings from GIM suggested the first eight weeks of coaching may be a critical period for eliciting behaviour change. Withdrawal rate was assessed as percentage of study participants who withdrew by the 2-, 6- and 12-month evaluation time points.

As part of the feasibility evaluation, we also measured usability and quality of the program. Each intervention group participant was asked to complete the Mobile App Rating Scale (MARS) [47] after their last online session. The 21-item MARS has four subscales that assess software-related Quality, Functionality, Information and Behaviour Change; responses to each are measured on a five-point Likert scale. Participants were also asked to complete relevant questions from the ‘Health Education Impact Questionnaire’ Version 3 (heiQ) about the quality of the program [48]. The questionnaire has nine items with responses ranging from 1 to 4: strongly disagree (1), disagree (2), agree (3) and strongly agree (4).

#### Primary short-term outcome measure: Skill and Technique Acquisition subscale of the Health Education Impact Questionnaire

Self-management skills were measured with the Skill and Technique Acquisition (STA) subscale of the heiQ, a widely used tool to measure the quality and outcomes of chronic disease self-management programs [49]. The heiQ has demonstrated high construct validity ranging from 0.70 to 0.83 for each of the dimensions and reliability >0.8 (48). It measures eight constructs by multi-item composite scales using a 4-point Likert scale: strongly disagree (1), disagree (2), agree (3) and strongly agree (4), with a mean score ranging from 1 to 4. The STA subscale has 4 items that aim to capture the knowledge-based skills and techniques that persons acquire (or re-learn) to help them cope with symptoms and health problems (e.g. ‘When I have symptoms, I have skills that help me cope’). It was chosen as the basis for the sample size calculation as skill building is a primary focus of the intervention [50].

#### Primary long-term outcome measure: cumulative days rehospitalised 12 months after baseline assessment

In an RCT by Gassaway et al. [30] evaluating a peer-mentoring self-management SCI program for patients receiving inpatient rehabilitation, cumulative days rehospitalised at 6 months after discharge from inpatient rehabilitation were significantly fewer for patients who received peer mentoring compared to controls (43% reduction, *p* < 0.001), with a mean rehabilitation length of stay of approximately 2 months. In our study, since participants were already living in the community and the intervention period was 6 months, the primary long-term outcome measure of days rehospitalised 12 months after baseline was calculated by summing the answer to the question ‘How many total nights did you spend in hospital in the past 6 months?’ at the 6-month and 12-month time points.

#### Secondary outcomes

In addition to the STA subscale, we planned to collect the other seven subscales of the heiQ: Health Directed Behaviour; Positive and Active Engagement in Life; Emotional Distress; Self-monitoring and Insight; Constructive Attitudes and Approaches; Social Integration and Support; and Health Services Navigation [48]. However, we noted considerable overlap in the content of three heiQ subscales. To reduce respondent burden and increase the probability of participants completing the measures, we did not collect three subscales: Health Directed Behaviour, Constructive Attitudes and Approaches and Social Integration and Support. We also collected the Secondary Conditions Scale, a 16-item self-report measure that targets secondary conditions associated with SCI that impact health [51], and the University of Washington Self-Efficacy Short Form, a 6-item self-report questionnaire rating confidence in self-management skills validated for the SCI population [52]. We measured Health-Related Quality of Life (HRQOL) using 3 questions from the International Spinal Cord Injury–Quality of Life (SCI QOL) basic dataset that rate satisfaction with general QOL, physical and psychological health [53] and the SCI-QOL Resilience Short Form, an 8-item measure of adaptation or adjustment after injury [54]. We also collected a measure of Social/Role Activities Limitations [55] and the 8-item Personal Health Questionnaire (PHQ) Depression Scale developed by the Stanford group to evaluate the CDSMP [56].

#### Descriptive variables and covariates

The following demographic and social characteristics were collected: age, gender, city/province of residence, language, employment status, education level, income level, marital status and living arrangement. Injury-related characteristics were also collected: time since injury; level of impairment and injury completeness; traumatic or non-traumatic; and primary mode of mobility. Given the nature of the intervention, at baseline we also collected the eHealth Literacy scale assessing perceived skills locating and applying information about health from the internet [57].

### Data collection

Data were collected at baseline and 2, 6 and 12 months after randomisation.

### Randomisation and blinding

A statistician prepared the group allocation schedule in advance of the study using an online tool. Blocking (block size of 4) was used to achieve an equal number of participants in each study group to maximize statistical efficiency [58]. The order of group assignment within the block was randomised. To ensure blinding of outcome assessment, a research assistant blinded to group assignment collected the quantitative data over the phone at 2, 6 and 12 months.

### Sample size calculation

The sample size for this pilot study was estimated based on findings from the developers of the heiQ, which provided benchmark estimates of change for each of the heiQ subscales. Using archived data from 2157 participants of chronic disease self-management programs conducted by a wide range of organisations in Australia between July 2007 and March 2013, they calculated percentile norms for individual heiQ scale scores and effect sizes for group change to assist managers, programme staff and clinicians of healthcare organisations to interpret their heiQ results [49]. Based on the Australian data, from baseline to 6-month follow-up, the effect size for the STA subscale was 0.50 (95%CI 0.45–0.55) [49]. With a target of 60 participants and >80% adherence, we will be able to provide a reasonable, bias-corrected estimate for a medium effect size for a future definitive RCT [59].

### Analysis

Feasibility outcomes were reported descriptively. Baseline data were reported using descriptive statistics. Continuous variables were summarized using means and standard deviations. Categorical variables were summarized using counts and percentages. When applicable, a total score was calculated for each scale by summing individual item scores. In cases where available, the T-score was calculated from the total score. Total scores and T-scores were summarized using means and standard deviations. Total scores at 6 months and 12 months were plotted against the value at baseline by treatment group. The Pearson correlation coefficient was calculated to quantify the relationship of the total score at 6 or 12 months with the baseline value. Analysis of covariance (ANCOVA) was used to estimate the effect of treatment on the scores while adjusting for the score value at baseline. Normally distributed outcomes were analyzed with ANCOVA models estimating the difference in outcome level at 6 and 12 months, controlling for baseline values. Treatment effects were reported with 95% confidence intervals (CI). Treatment effect can be interpreted as the difference in score among persons in the treatment group versus those in the control group after adjusting for baseline score. This method was used for all continuous outcomes.

The distribution of counts for the days hospitalised was both zero-inflated and over-dispersed, which severely limited analytical approaches that can be used beyond descriptive statistics. Due to small counts, a Fisher’s exact test was used to compare the numbers between groups. Because of the outliers, a non-parametric test for a difference in medians was performed.

## Results

### Trial feasibility

#### Recruitment

**Fig. 1:**
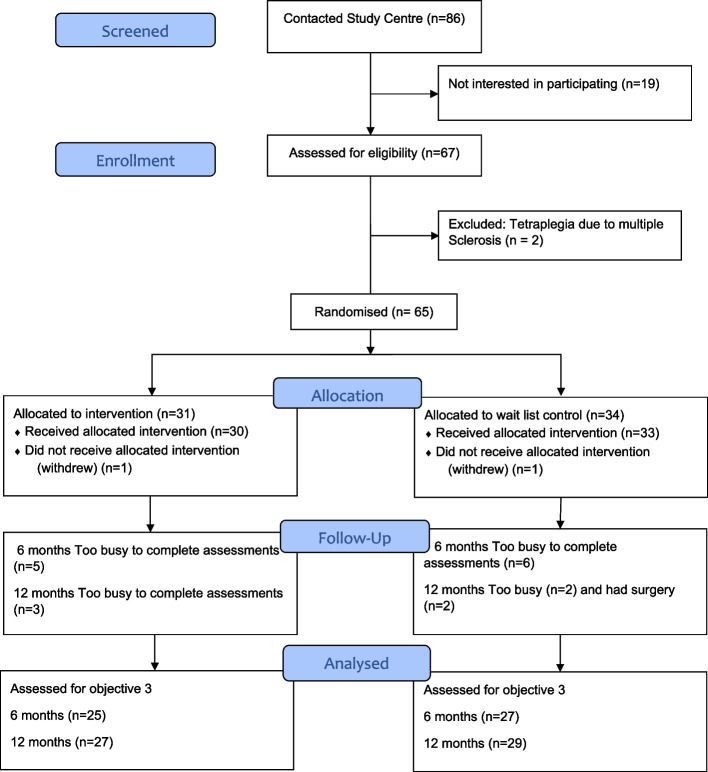
CONSORT flow diagram

Figure 1 describes the flow of participants through the trial. Recruitment occurred between September 2019 and September 2020. Eighty-six individuals inquired about the study, 67 were screened for eligibility and two were excluded because their tetraplegia was due to multiple sclerosis and not SCI. Of 65 participants, 31 were allocated to the intervention and 34 to the waitlist control group; two participants withdrew, leaving 30 intervention and 33 controls. Participants remained in the group to which they were assigned.

Participants were recruited from the community through advertisements in SCI consumer organization newsletters and websites, peer health coach networks and clinicaltrials.gov trial registration. No study participants were recruited from rehabilitation hospitals that have SCI units in Ontario, BC and Saskatchewan, due to COVID-19 restrictions. Feasibility of recruitment was to be assessed by the number and proportion of consenting individuals per month benchmarked against whether 66 participants could enter the trial in an 8-month recruitment period. This was determined to not be a useful measure given the impact of COVID-19 restrictions, which extended the recruitment period to 12 months.

#### Respondent characteristics

Baseline demographic, social and injury characteristics of participants in intervention and waitlist control groups are presented in Table 2. Mean age of participants in the intervention group was 49.6 years compared to 48.7 years in the control group. Mean time since injury was 25.6 years among participants in the intervention and 20.2 years in the control group. This was a highly educated group, with only 7/63 (11%) participants reporting having high school or less education. There were no significant differences with respect to the amount of missing data across variables at any time point. The only baseline difference of note is that there were more participants with cervical injuries in the control group (19 versus 8 intervention) and more participants with thoracic/lumbar injuries in the intervention group (18 versus 8 control).

**Table 2:** Baseline and injury characteristics of participants, reported as *n* and percentage, unless otherwise indicated

Characteristic		Intervention (*N* = 30)	Control (*N* = 33)
Age (years)	Mean (SD)	49.6 (11.1)	48.7 (14.1)
	Median (Minimum, Maximum)	49 (21, 76)	50 (29, 72)
	Missing	2	7
Time since injury (years)	Mean (SD)	25.6 (13.3)	20.2 (13.5)
	Median (Minimum, Maximum)	24 (3, 58)	16 (1, 55)
	Missing	4	4
Gender	Male	17 (57)	13 (43)
	Female	13 (43)	17 (57)
	Transgender/Gender neutral	0	0
	Missing	0	3
Primary language	English	27 (96)	26 (93)
	French	1 (4)	0 (0)
	Other	0 (0)	2 (7)
	Missing	2	5
Province	British Columbia	16 (53)	23 (70)
	Ontario	7 (23)	4 (12)
	Saskatchewan	5 (17)	4 (12)
	Other	2 (7)	2 (6)
	Missing	0	0
Employment	Employed full-time	3 (12)	7 (24)
	Employed part-time	3 (12)	5 (17)
	Unemployed	19 (76)	17 (59)
	Missing	5	4
Highest level of education	High school or less	3 (12)	4 (15)
	College/Bachelor’s degree	16 (64)	19 (70)
	Postgraduate	6 (24)	4 (15)
	Missing	5	6
Marital status	Single/Never married	9 (35)	11 (39)
	Married/Partnered	11 (42)	10 (36)
	Separated/Divorced/Widowed	6 (23)	7 (5)
	Missing	4	5
Living arrangement	Living alone	7 (26)	11 (38)
	Living with someone	17 (63)	15 (52)
	Other	3 (11)	2(7)
	Missing	3	4
eHealth literacy	Mean (SD)	30.6 (5.1)	29.9 (5.1)
	Median (Minimum, Maximum)	30.5 (22.0, 40.0)	31.0 (20.0, 40.0)
	Missing	2	5
Injury characteristics	Paraplegic	18 (69)	13 (50)
	Quadriplegic	8 (31)	13 (50)
	Missing	4	7
	Traumatic	21 (78)	21 (81)
	Non-traumatic	5 (19)	4 (15)
	Other	1 (4)	1 (4)
	Missing	3	7
	Complete	12 (44)	8 (31)
	Incomplete	15 (56)	18 (69)
	Missing	3	7
	Cervical	8 (29)	19 (66)
	Thoracic	12 (43)	7 (24)
	Lumbar/Sacral	6 (22)	1 (3)
	Other	2 (7)	2 (7)
	Missing	2	4
Mobility	Manual wheelchair	16 (59)	12 (41)
	Powered wheelchair	7 (26)	11 (38)
	Walker/Cane/Other	4 (14)	6 (21)
	Missing	3	4

*SD* standard deviation

#### Retention

Of 30 intervention and 33 control participants who received the allocated intervention, 25 (83%) intervention and 29 (88%) control participants provided data at 6 months, and 27 (90%) intervention and 29 (88%) control participants provided data at 12 months. Remuneration (up to $300 CDN) was initially to be paid at the end of the study, but to incentivize participants to complete assessments we provided remuneration ($100 CDN per completed assessment) in the form of a gift card of their choice (grocery, drug store, department store) after they completed each of the assessments at baseline, 6 and 12 months. The overall retention rate at 12 months was 89% (56/63).

#### SCI&U program delivery

The expectation was to match coaches and participants with similar characteristics. To facilitate matching, we created coach profiles for the platform. Matching with respect to the level of impairment was not possible, as all but one of the coaches had higher level injuries and required wheelchairs.

It was anticipated that follow-up data would be collected at 2 months based on previous research on behaviour change for physical activity in an SCI population, which suggested that the first 2 months of coaching may be a critical period for eliciting behaviour change [46, 60]. All online sessions for SCI&U were expected to be completed within 6 months, with the majority being completed after 2 months. However, intervals between sessions were allowed to vary to promote flexibility. Intervals were decided collaboratively between coach and client and ranged between 1 and 2 weeks. Thus many participants were still actively involved in the intervention and did not have the majority of their sessions completed at the 2-month data collection point, so part way through the trial we decided to forego collection of 2-month data.

The mean ± SD number of coaching sessions was 12.6 ± 2.3 (median 13), and individual sessions lasted 55 ± 26 (median 57) minutes. Only one participant completed less than 8 coaching sessions.

Among sessions annotated with a topic within the coaching platform, the most common topics discussed were aging with an SCI (13% of themed sessions), pain, exercise, mental health and diet (each 10–11% of themed sessions), bladder and skin. In almost 30% of sessions, coaches and participants did not pre-select a scripted topic to discuss. As the pandemic progressed, COVID-19 also became a topic (3% of themed sessions).

#### Usability and quality of the program

Twenty-five of 30 (83%) participants in the intervention group completed the MARS and the heiQ course quality questions after their last coaching session. Mean scores ± SD on the MARS were 3.9 ± 0.8 for software functionality, 3.2 ± 0.7 for quality, 4.2 ± 0.7 for behaviour change and 4.2 ± 0.7 for information. Users assigned the service 4 out of 5 stars for overall quality, on average, yet indicated program fees would be a potential barrier to program adoption. Mean scores for the course quality questions of the heiQ ranged from 3.5 to 4.0.

### Primary short-term outcome: Skill and Technique Acquisition subscale of the heiQ

Table 3 provides the raw scores (i.e. T-scores) on primary and secondary outcomes by intervention group. Table 4 provides estimates of effect sizes of the intervention on the heiQ subscale scores at 6 and 12 months, adjusted for subscale scores at baseline. After adjusting for baseline subscale score, the difference in 6-month STA subscale score between persons in the treatment group compared to those in the control group was 0.56 (95% CI: -0.41, 1.52). The difference in 12-month score between persons in the treatment group compared to those in the control group was 0.72 (95% CI: -0.28, 1.72). An effect size of 0.5 is the benchmark for change in STA [49].

**Table 3:** Raw scores or T-scores for outcome variables for intervention and control groups at baseline, 6 months and 12 months follow-up, reported as mean ± standard deviation

	Baseline		6 Months		12 Months	
	Intervention (*N* = 28)	Control (*N* = 29)	Intervention (*N* = 25)	Control (*N* = 27)	Intervention (*N* = 27)	Control (*N* = 29)
*heiQ Subscales*						
Skill and Technique Acquisition	2.9 ± 0.5	3.0 ± 0.5	3.2 ± 0.6	3.0 ± 0.5	3.2 ± 0.6	3.0 ± 0.4
Self-monitoring and Insight	3.0 ± 0.7	3.3 ± 0.4	3.3 ± 0.4	3.2 ± 0.4	3.4 ± 0.5	3.2 ± 0.5
Emotional Distress	2.6 ± 0.8	2.2 ± 0.7	2.4 ± 0.9	2.2 ± 0.7	2.4 ± 0.9	2.3 ± 0.9
Health Services Navigation	3.0 ± 0.7	3.1 ± 0.5	3.2 ± 0.6	3.2 ± 0.6	3.2 ± 0.6	3.2 ± 0.4
Positive and Engagement in Life	3.0 ± 0.7	3.2 ± 0.5	3.1 ± 0.6	3.1 ± 0.6	3.1 ± 0.6	3.1 ± 0.5
*Other Outcome Variables*						
Secondary Conditions Scale	19.5 ± 9.6	14.6 ± 7.8	17.2 ± 10.3	17.6 ± 10.2	14.1 ± 6.5	15.0 ± 7.8
University of Washington Self-Efficacy Scale T-score	44.2 ± 11.5	46.7 ± 9.1	47.7 ± 10.8	46.8 ± 10.1	46.5 ± 12.2	47.3 ± 9.4
Health Related Quality of Life	19.2 ± 8.6	20.2 ± 7.7	21.6 ± 8.6	21.0 ± 7.9	21.4 ± 7.7	21.4 ± 6.5
SCI QoL Resilience	47.4 ± 7.7	50.3 ± 8.2	49.0 ± 8.3	49.6 ± 7.1	49.7 ± 10.9	49.8 ± 7.8
Social/Role Limitations	8.0 ± 5.4	6.7 ± 5.8	6.3 ± 5.6	6.5 ± 5.4	6.7 ± 4.8	6.3 ± 5.4
PHQ-8 Patient Depression Questionnaire	8.4 ± 6.3	5.8 ± 4.5	6.5 ± 5.2	5.4 ± 4.4	7.2 ± 6.1	5.6 ± 4.3

*heiQ* Health Education Impact Questionnaire, *SCI* spinal cord injury, *QoL* Quality of Life, *PHQ* Personal Health Questionnaire

**Table 4:** ANCOVA treatment effect estimates at 6 months and 12 months follow-up for outcome variables

Outcome variable	6 months follow-up			12 months follow-up		
	Estimate	95% CI	*p* value	Estimate	95% CI	*p* value
*heiQ Subscales*						
Skill and Technique Acquisition	0.56	-0.4, +1.5	0.25	0.72	-0.3, 1.7	0.15
Self-Monitoring and Insight	0.94	-0.2, +2.1	0.11	1.51	+0.3, 2.7	0.01
Emotional Distress	-0.45	-2.0, +1.1	0.56	-1.40	-3.0, 0.2	0.09
Health Services Navigation	0.23	-1.0, +1.5	0.71	0.43	-0.8, 1.6	0.46
Positive and Active Engagement in Life	-0.005	-0.9, +0.9	0.99	0.15	-0.9, 1.2	0.78
*Other Outcome Variables*						
Secondary Conditions Scale	-0.40	-3.4, +2.7	0.79	-1.5	-4.3, 1.4	0.31
University of Washington Self-Efficacy Scale	1.85	-2.4, +6.1	0.39	0.83	-3.5, 5.2	0.71
Health Related Quality of Life	-0.78	-2.8, +1.3	0.45	-0.46	-2.5, 1.6	0.66
SCI QoL Resilience	0.03	-2.0, +2.0	0.98	0.88	-1.8, 3.5	0.51
Social/Role Limitations	-0.78	-2.8, +1.3	0.45	-0.46	-2.5, 1.6	0.66
PHQ-8 Patient Depression Questionnaire	-0.06	-2.3, +2.2	0.96	-0.20	-2.4, 2.0	0.85

*heiQ* Health Education Impact Questionnaire, *SCI* spinal cord injury, *QoL* quality of life, *PHQ* Personal Health Questionnaire

### Primary long-term outcome measure: cumulative days rehospitalised in the 12 months following baseline assessment

At 6 and 12 months, data points were missing for 7 and 5 participants in the intervention group, respectively, compared to 13 and 12 participants in the waitlist control group, respectively (Table 5). Most participants at 12 months (17 intervention and 20 controls) had zero nights in hospital. Among the 9 persons for whom the information was available and who had spent at least one night in hospital, the median number of nights in hospital in the past 12 months was 11 (95% CI: 3, 19) nights in the intervention group (*n* = 5) and 24 [5–95] nights in the control group (*n* = 4).

**Table 5:** Cumulative days rehospitalised 6 and 12 months after baseline assessment

Cumulative days rehospitalised		Intervention (*N* = 30)	Control (*N* = 33)
6 months after baseline assessment	Mean (Standard Deviation)	1.5 (4.3)	4.3 (19.0)
	Median (Minimum, Maximum)	0 (0, 15)	0 (0, 85)
	Missing	7	13
12 months after baseline assessment	Mean (Standard Deviation)	1.9 (5.2)	6.6 (21.1)
	Median (Minimum, Maximum)	0 (0, 19)	0 (0, 95)
	Missing	5	12

There was no difference in rehospitalisation rates between groups at either 6- or 12-months post-intervention. Because most participants did not experience rehospitalisation, a secondary analysis comparing the proportion of persons in each group who experienced any rehospitalisations also found no difference in rates of rehospitalisation at 6 months (13% intervention versus 10% control) and at 12 months (20% intervention versus 19% control).

### Secondary outcome measures

Of the other four heiQ subscales, Self-Monitoring and Insight had a significant treatment effect at 12 months (*p* = 0.01; Table 4). This construct captures the individuals’ ability to monitor their condition and their physical and/or emotional responses that lead to insight and appropriate actions to self-manage (e.g. “I carefully watch my health and do what is necessary to keep as healthy as possible”). The difference in 12-month score between persons in the intervention group compared to those in the control group was 1.51 (95% CI: 0.39, 2.69). At 12 months follow-up, a greater proportion of the intervention group strongly agreed with the statements in the Self-Monitoring and Insight construct compared with the control group (Table 6).

**Table 6:** Number and percentage of respondents in intervention and control groups at 12 months follow-up who strongly agree with each of the individual items on the Self-Monitoring and Insight scale

Item on Self-Monitoring and Insight Scale	Intervention (*N* = 27) *N* (%) strongly agree at 12 months follow-up	Control (*N* = 29) *N* (%) strongly agree at 12 months follow-up
I have realistic expectations of what I can and cannot do	10 (37%)	7 (24%)
I regularly monitor changes in my health	16 (59%)	8 (28%)
I know what things can trigger my health problems and make them worse	16 (59%)	8 (28%)
When I have health problems, I have a clear understanding of what I need to do to control them	16 (59%)	7 (24%)
I have a very good understanding of when and why I am supposed to take my medication	14 (52%)	4 (14%)
I carefully watch my health and do what is necessary to keep as healthy as possible	19 (70%)	16 (55%)

The other heiQ subscale with a large effect was Emotional Distress, with an effect estimate of -1.40 (95% CI: -3.04, 0.23) at 12 months (Table 4); this difference was not significantly different (*p* = 0.09). Minimal changes were observed for the subscale Positive and Active Engagement in Life and for Health Services Navigation at 12 months.

The other secondary outcomes demonstrated no notable differences between the control and intervention groups (Table 4).

## Discussion

This study examined the feasibility of conducting a definitive RCT to determine the effectiveness of an online self-management program using peer health coaches to improve self-management skills and reduce rehospitalisations for persons living with SCI. We found that, overall, the trial methodology and procedures were feasible, and the intervention was acceptable to participants. Several challenges identified in this feasibility trial are being changed to improve delivery of the intervention and trial methodology in the definitive trial (Table 7).

**Table 7:** Changes to the intervention and trial methodology for definitive randomised trial

Problem identified during the feasibility trial	Change made for the definitive trial
Could not recruit from inpatient rehabilitation hospitals due to pandemic restrictions	Have focused recruitment efforts for inpatient rehabilitation hospitals
Participants who enrolled in study were many years post-injury	Increase recruitment efforts to identify participants within 1–5 years of injury
There were more participants with cervical injuries in the control group and more participants with thoracic/lumbar injuries in the intervention group	Consider block randomisation
Some participants had incomplete injuries and were not wheelchair users, but all but one coach was a wheelchair user	Have a mix of coaches who are ambulatory or wheelchair users
Participants did not follow the proposed timing for the online coaching sessions—one-half of coaching sessions were expected to be completed within 2 months	All online sessions are expected to be completed within 6 months, but intervals between sessions will be allowed to vary in order to promote flexibility. Drop 2-month follow-up data collection
Online coaching sessions are expected to cover a health-related topic (e.g. bladder, bowel, skin, pain, healthy eating, physical activity or stress, anxiety and depression, etc.) and a self-management skill topic (e.g. action planning, goal setting, problem solving, mood management, navigating the health care system, communicating with health care providers)	The selection of topics and the order in which they are addressed will be determined by the study participant, with input from their peer health coach when requested. To ensure we meet the unique needs of participants, the coach and participant will determine jointly how many sessions to spend on a topic
Sometimes had to use telephone for coaching due to a lack of high-speed internet connection among participants	Telephone option for coaching program will be available
Too many outcome measures to complete	Limit outcome measures to only those that showed potential for change
MARS scale may be too long for definitive trial	Replace MARS with the 10-item System Usability Scale [61]
Some participants reported struggling with the technical skills required to navigate the platform to complete the outcome measures	Have a research assistant help participants with completion of outcome measures to ensure completeness and quality
Largest effect sizes were for two heiQ subscales: Emotional Distress, and Self-Monitoring and Insight	Consider Emotional Distress and Self-Monitoring and Insight as primary outcomes
Wanted same distribution of males and females in intervention and control groups	Stratify by sex
Hospitalisation data skewed	Data on hospitalisations will not be collected

The majority of this pilot trial was conducted during the COVID-19 pandemic when indoor mask use was mandatory and restrictions were in place for nonessential travel, social gatherings and businesses. The COVID-19 pandemic forced Canadians with SCI to adapt to a new level of physically distant health care, led to reduced access to numerous health care services and increased self-isolation to prevent the spread of infection [62, 63]. The pandemic had major implications for recruitment sources, characteristics of study participants and outcomes for this pilot trial. We could not recruit from rehabilitation hospitals and had to rely on community-based SCI organizations to place ads in their newsletters and on their websites. Consequently, some participants were older than expected. They may have wanted a coach because they were lonely due to pandemic restrictions; this was supported by the higher values they scored on the emotional distress scale. Because participants were older, they already had some self-management skills, which might explain why self-monitoring and insight had more change in this study. Finally, hospitalization data was affected because participants were less likely to go to the Emergency Department or have a hospital stay during the pandemic.

Individuals with SCI and mobility challenges prefer web-based programming over in-person interventions [24, 25], as it simulates a face-to-face interaction and limits the need for in-person visits [33, 34]. Telehealth interventions were swiftly adopted during the pandemic and more than doubled, from 9.9% to 25.4%, due to the need for patients with SCI to safely access care [62]. This might explain the acceptability of our program, as there were no concerns or health risks involved with traveling to appointments with an online program. In addition, participation in the coaching sessions was high, with a mean of 12.6 sessions out of a possible maximum of 14 sessions; perhaps the program was a way for patients to address social isolation.

In the current pilot trial study, the five most common topics discussed were aging with an SCI, pain, exercise, mental health and diet. In a recent needs assessment of 38 individuals with SCI who were primarily White (89.5%), male (63.2%), an average age of 47.2 years and the majority more than 10 years out from injury, participants expressed that a self-management program would help them feel less alone and that their ‘cries of help’ would be heard [64]. In that study, with respect to topics, participants indicated that psychological health and coping was most important followed by pain, spasticity, and aging with SCI. These topic priorities are similar to our results.

One of the outcome measures that had the most change in the current pilot trial was the heiQ subscale Emotional Distress. To this end, fear and anxiety of contracting COVID-19 and perceived vulnerability may be further contributors to worsening mental health. In the Mesa et al. [62] post-COVID study of individuals with SCI living in the community in BC, Canada, more than one-third of survey respondents reported probable depression. This rate of probable depression was greater than the rates found in the SCI population in other recent pre-pandemic studies [65].

We were not able to recruit from outpatient clinics in the rehabilitation hospitals, which were closed during the pandemic and visits were either virtual or by telephone. We had to rely on community sources. Participants were, on average, 25 years post-injury. At baseline, on the STA scale, which aims to capture the knowledge-based skills and techniques persons acquire (or re-learn) to help them cope with symptoms and health problems [50], over 80% of intervention and control subjects agreed or strongly agreed that they had the skills to self-manage. For example, they reportedly had skills to help cope when symptoms arose, a very good idea of how to manage health problems, effective ways to prevent symptoms and a good understanding of equipment needed to make life easier. This may explain why there was no difference in self-management skills between the intervention and control groups at 12 months, as this group may have already learned how to manage their SCI given how long they were living with the injury. We did, however, note differences in self-monitoring and insight, which captures individuals’ ability to monitor their condition, and the physical and/or emotional responses that lead to insight and appropriate actions to self-manage. When we examined the responses to the individual items at 12-month follow-up, a greater proportion of the intervention group strongly agreed to the statements in the self-monitoring and insight construct (Table 7). This finding suggests that self-management support to promote self-monitoring and insight may be more appropriate than skill development for a group who has been managing their injury for a long time.

For the definitive trial, it may be important to recruit individuals who are newly injured, as they often describe feeling unprepared for returning home, physically and psychologically [66, 67]. After returning home, people often experience isolation, depression and low levels of physical and psychosocial functioning, coupled with a perception of system abandonment, claiming the transition to be like “falling off a cliff” [16]. Major depressive disorder occurs most commonly 1 to 5 years post-SCI, and approximately one-third of individuals have mental health problems that perpetuate into individuals’ lives even after 5 years following discharge [68, 69]. The limited time for provision of health education and skill acquisition in the inpatient setting means that individuals with SCI are entering the community with deficits in knowledge and fewer self-management skills to enable successful community re-integration [70]. To reach more recently injured individuals for the definitive trial who are more likely to benefit from a self-management program, recruitment methods will include both rehabilitation hospitals, where we anticipate challenges due to the pandemic will no longer be an issue, and through SCI consumer organisations who provide peer support services. This pilot study also underscores the need to consider the issue of health literacy and not just eHealth literacy and how to assess it in this population.

### Limitations

Participants were not blinded to their group allocation, which could have resulted in a bias in the reporting of numerous self-report measures. The effects observed may be influenced by the study being conducted during the COVID-19 pandemic. Also, generalizability of the findings is limited to individuals with SCI who were many years post-injury. Thus, the treatment effect observed for self-monitoring and insight could be spurious and will need to be confirmed. Additionally, participants were almost all White and well educated, so we are unable to comment on the applicability of the intervention and/or trial procedures for individuals with different ethnic and educational backgrounds. Participants commented that there were too many outcome measures to complete. Finally, hospitalisation data were difficult to interpret and difficult for participants to recall; results may have been affected by various COVID-19 policies.

## Conclusions

The findings of this pilot randomised trial suggest that it was possible to achieve recruitment and retention targets for the SCI&U online peer health coaching program even during the COVID-19 pandemic. However, it was difficult to recruit individuals with recent SCI, i.e. within 5 years of injury. Overall, the SCI&U platform was assessed as having good usability and the program being of high quality. This pilot study demonstrated that SCI&U had a medium effect on skill and technique acquisition, had a large effect on reducing emotional distress and significantly improved self-monitoring and insight among a group of participants who were on average over 20 years post-injury. Given that most of the trial was conducted during the COVID-19 pandemic, these results need to be confirmed in a definitive trial, and further research is needed to determine the impact of the SCI&U peer health coaching program on those living with recently acquired SCI.

## Data Availability

The datasets generated and/or analysed during the current study are not publicly available due to small numbers and possible identification of individuals, but are available from the corresponding author on reasonable request.

## Abbreviations

ANCOVA: Analysis of covariance
BAP: Brief Action Planning
BC: British Columbia
CDSMP: Chronic Disease Self-Management Program
CI: Confidence interval
CONSORT: Consolidated Standards of Reporting Trials
GIM: Get In Motion
heiQ: Health Education Impact Questionnaire
HRQOL: Health-related quality of life
MARS: Mobile App Rating Scale
MCMC: My Care My Call
PHQ: Personal Health Questionnaire
RCT: Randomised controlled trial
SCI: Spinal cord injury
SCI QOL: Spinal Cord Injury–Quality of Life
SCI&U: Spinal Cord Injury and You
STA: Skill and Technique Acquisition
